# Multiple Choice Neurodynamical Model of the Uncertain Option Task

**DOI:** 10.1371/journal.pcbi.1005250

**Published:** 2017-01-11

**Authors:** Andrea Insabato, Mario Pannunzi, Gustavo Deco

**Affiliations:** 1 Universitat Pompeu Fabra, Center for Brain and Cognition, Barcelona, Spain; 2 Institució Catalana de Recerca i Estudis Avançats (ICREA), Barcelona, Spain; Oxford University, UNITED KINGDOM

## Abstract

The uncertain option task has been recently adopted to investigate the neural systems underlying the decision confidence. Latterly single neurons activity has been recorded in lateral intraparietal cortex of monkeys performing an uncertain option task, where the subject is allowed to opt for a small but sure reward instead of making a risky perceptual decision. We propose a multiple choice model implemented in a discrete attractors network. This model is able to reproduce both behavioral and neurophysiological experimental data and therefore provides support to the numerous perspectives that interpret the uncertain option task as a sensory-motor association. The model explains the behavioral and neural data recorded in monkeys as the result of the multistable attractor landscape and produces several testable predictions. One of these predictions may help distinguish our model from a recently proposed continuous attractor model.

## Author Summary

Recently many studies began to investigate the brain signature of complex cognitive functions such as decision confidence, the feeling of certainty/uncertainty associated with a decision. To this aim, the uncertain option task has been widely adopted in order to assess the confidence in animals. In this study we present a model, detailed at the neuron and synapse level, able to account for the behavior of animals in this task. In addition our model is able to reproduce the neural dynamics found in monkeys brain during this task. However our model is only equipped with a simple multiple choice mechanism and has no mechanism devoted to calculate the confidence. Therefore our study support the idea that the uncertain option task can be solved without relying on confidence assessment (metacognition). The model is based on the idea that the neural dynamics fluctuates around stable equilibrium points (attractors) and associates the landscape of these attractors with the behavior of the monkeys. Finally, our model makes several predictions that could be easily tested in a new experiment. One of these predictions may help distinguish our model from a different one that has been recently proposed.

## Introduction

Decision confidence, a simple form of metacognition, has been receiving increasing attention in neuroscience over the last ten years. Many studies began to unravel possible neural correlates of confidence both in rodents [1, 2] and in primates [3–5]. At the same time several models have been proposed to account for behavioural and neural signatures of decision confidence [3, 6–9]. In order to study confidence in animals, several experimental tasks have been used, which can be categorized in: post-decision wagering [1], uncertain option [10], pre-decision wagering [11] and information seeking [12]. In this article we focus on the uncertain option task. More specifically we will concentrate on the behavioural and neural data by [3]. In this experiment [3] the subjects have to decide about the prevalent direction of motion of a random dots display. In each trial the subjects are presented the choice between e.g. “left” category, “right” category and a third option (called “sure” target). Correct choices lead to reward, error choices lead to a penalty time out and the sure option yields a smaller but sure reward. These trials are usually called “free choice”, since the subject is allowed to escape by choosing the sure target, compared to forced choice binary tasks. The rationale of this task is that animals should choose the uncertain option when they are less confident in the decision and therefore prefer to avoid a probable punishment. The neural activity in lateral intraparietal sulcus (LIP) was recorded while the subjects were performing the task.

While some abstract models have already been proposed for the uncertain option task [3, 13–15], last year a biologically detailed model has been proposed [9] based on a continuous representation of motion direction. In this article we propose a different modelling approach. Instead of using a continuous representation we associate one neural pool to each possible choice: left, right and sure. As a consequence, our model is a biologically plausible implementation of a discrete multiple-choice decision mechanism [16]. The underlying hypothesis of our work is that the uncertain option task can be solved as a perceptual decision between three alternatives with a delayed stimulation protocol. Our model accounts for both behavioral and neural data [3].

We characterize the dynamics of the model in terms of the underlying multistable attractor landscape and investigate the influence of sensory evidence on the dynamics. In addition we study the modification of the attractor landscape induced by changes in input intensity and link such modification of the attractor landscape with the choice behavior of the network. This analysis shows that multistability, particularly near a bifuraction is needed to reproduce experimental data. Finally, we show three experimental predictions to falsify or verify our model. 1. Only the maximum activity of decision neurons selective for right and left choices is determining the probability of a sure response. 2. The probability of sure target choices separated in correct and error trials presents an X-pattern. 3. The decision times of the model have a bimodal distribution. Interestingly prediction 1 may offer a way to distinguish the continuous attractor network [9] and our discrete attractors network, since the continuous attractor model seems to make a different prediction.

## Materials and Methods

### Model details and mean-field reduction

**Fig 1:**
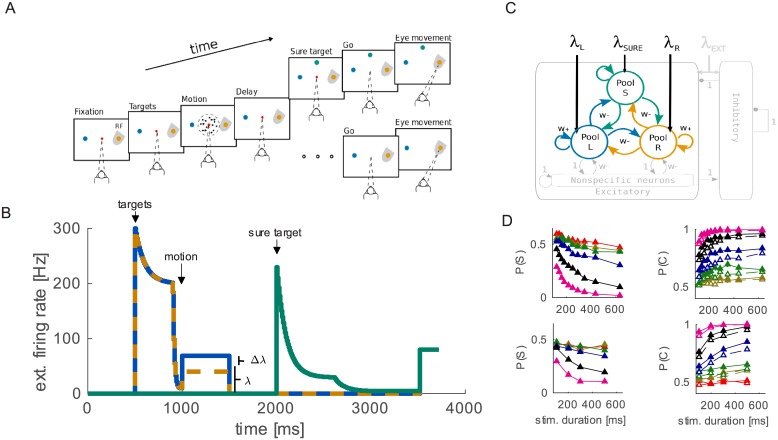
Task description, psychophysics and model structure. (A) Experimental task procedure [3]. (B) Time course of input. Input to pool R, L and S is represented respectively by orange, dashed blue and green line (colors are consistent with B). A first spontaneous phase is followed by the onset of the target that elicit a strong input with temporal adaptation. At *t* = 1 s the motion input is turned on: pool L receives *λ*_*L*_ = *λ* − Δ*λ* and pools R receives *λ*_*R*_ = *λ* + Δ*λ*. After motion input, during the delay phase, the network only receives background noise. In free choice trials, pool S receives input due to the onset of the sure target 500 ms after the offset of the motion input. (C) Schematic representation of the attractor network model. Arrowhead connections represent excitatory projections, dot-head connections represent inhibitory projections. Arrows coming from outside represent external inputs. (D) Psychophysics data measured experimentally (top row) and produced by the model (bottom row). The model reproduces qualitatively the effect of both stimulus duration and coherence on the probability of correct responses in forced choice trials (open circles, right panel), as well as on the probability of choosing the “sure” option (left panel). Moreover the model correctly predicts the increased P(*correct*) in free choise trials (filled circles, right panel).

We run simulations of 1000 trials for each stimulus condition (duration and coherence or Δ*λ*). The input to the network is modeled as Poisson spike trains coming from 800 external neurons. The stimulation protocol of the selective pools R and L is the following. First during 500 ms the pools only receives the background activity (3 Hz from each external neuron) to assure that the network is in resting state. After the resting phase the target input produces a strong response in decision pools L and R. The target input is modeled following two exponential decays (we implemented the same target input as in [16], where more details can be found). The target input lasts for 500 ms. After the target phase the stimulus input is turned on: this input is modeled as *λ* + Δ*λ* for pool L and *λ* − Δ*λ* for pool R. Parameter *λ* represent the average intensity of input common to both L and R pools. Parameter Δ*λ* represents the evidence for the decision, i.e. the motion coherence of the RDM stimulus. We simulated for duration of the stimulus: 100, 200, 300 and 500 ms. After stimulus offset *λ* and Δ*λ* are set to 0 until the end of the trial. At the end of the trial a saccade related signal of 80 Hz is delivered to pools L, R and S for 100 ms. This input has the only effect to increase the firing rate at the end of the trial but has no functional meaning in the model. We implemented it following previous modelling studies of LIP decision neurons (see e.g. [16]). The pool S receives only the background activity until the “sure” target is turned on. In forced choice trials there is no sure target and pool S only receives the background activity throughout the trial. In free choice trials the sure target is turned on(500 ms after the end of the RDM stimulus. The input due to the “sure” target is modeled in the same way as the target input to pools R and L (see [16] for details) and lasted until the end of the trial; the decay of this input has a higher asymptote at 5 Hz, compared to the target input that decays until 0. This input does not depend on other model parameters (e.g. *λ* or Δ*λ*). The stimulation protocol is shown in Fig 1B.

Detailed description of the neuron and synapse model is given in Tables 1 and 2.

**Table 1:** Model summary. Parameters values are given in Table 2.

**A**		**Model Summary**	
**Populations**		five	
**Connectivity**		full, no synaptic delay	
**Neuron model**		Leaky Integrate-and-Fire, fixed threshold, fixed refractory time	
**Synapse model**		Instantaneous jump and exponential decay for AMPA and GABA and exponential jump and decay for NMDA receptors	
**Plasticity**		-	
**Input**		Independent fixed-rate Poisson spike trains to all neurons	
**Measurements**		Spike activity	
**B**		**Populations**	
Total number of neurons	*N* = 1000		
Excitatory neurons in each module		*N*_*E*_ = 0.8 ⋅ *N*	
Inhibitory neurons in each module		*N*_*I*_ = 0.2 ⋅ *N*	
**Name**	**Size**	**Name**	**Size**
L (decision “left”)	*N*_*L*_ = *f* ⋅ *N*_*E*_	Nonspecific	*N*_*E*_ − 3*N*_*A*_
R (decision “right”)	*N*_*R*_ = *f* ⋅ *N*_*E*_	Inhibitory	0.2 ⋅ *N*
S (decision “sure target”)	*N*_*S*_ = *f* ⋅ *N*_*E*_		
**C**		**Neuron and Synapse Model**	
**Type**		Leaky integrate-and-fire, conductance-based synapses	
**Subthreshold dynamics**		$C_{m}\dot{V}\left(t\right)=-g_{L}\left(V\left(t\right)-V_{L}\right)-I_{AMPA,ext}\left(t\right)-I_{AMPA,rec}\left(t\right)-I_{NMDA}\left(t\right)-I_{GABA}\left(t\right)$	
**Synaptic currents**		$I_{AMPA,ext}\left(t\right)=g_{AMPA,ext}\left(V\left(t\right)-V_{E}\right)\sum_{j=1}^{N_{ext}}s_{j}^{AMPA,ext}\left(t\right)$	
		$I_{AMPA,rec}\left(t\right)=g_{AMPA,rec}\left(V\left(t\right)-V_{E}\right)\sum_{j=1}^{N_{E}}w_{j}s_{j}^{AMPA,rec}\left(t\right)$	
		$I_{NMDA}\left(t\right)=\frac{g_{NMDA}\left(V\left(t\right)-V_{E}\right)}{1+\left[Mg^{2+}\right]exp\left(-0.062V\left(t\right)\right)/3.57}\times \sum_{j=1}^{N_{E}}w_{j}s_{j}^{NMDA}\left(t\right)$	
		$I_{GABA}\left(t\right)=g_{GABA}\left(V\left(t\right)-V_{I}\right)\sum_{j=1}^{N_{I}}w_{j}s_{j}^{GABA}\left(t\right)$	
**Fraction of open channels**		$\frac{ds_{j}^{AMPA,ext}\left(t\right)}{dt}=-\frac{s_{j}^{AMPA,ext}\left(t\right)}{\tau_{AMPA}}+\sum_{k}\delta \left(t-t_{j}^{k}\right)$	
		$\frac{ds_{j}^{AMPA,rec}\left(t\right)}{dt}=-\frac{s_{j}^{AMPA,rec}\left(t\right)}{\tau_{AMPA}}+\sum_{k}\delta \left(t-t_{j}^{k}\right)$	
		$\frac{ds_{j}^{NMDA}\left(t\right)}{dt}=-\frac{s_{j}^{NMDA}\left(t\right)}{\tau_{NMDA,decay}}+\alpha x_{j}\left(t\right)\left(1-s_{j}^{NMDA}\left(t\right)\right)$	
		$\frac{dx_{j}^{NMDA}\left(t\right)}{dt}=-\frac{x_{j}^{NMDA}\left(t\right)}{\tau_{NMDA,rise}}+\sum_{k}\delta \left(t-t_{j}^{k}\right)$	
		$\frac{ds_{j}^{GABA}\left(t\right)}{dt}=-\frac{s_{j}^{GABA}\left(t\right)}{\tau_{GABA}}+\sum_{k}\delta \left(t-t_{j}^{k}\right)$	
**Spiking**		if *V*(*t*) ≥ *V*_*θ*_ ∧ *t* > *t*^⋆^ + *τ*_*rp*_	
		1. *t*^⋆^ = *t*	
		2. emit spike at time *t*^⋆^	
		3. *V*(*t*) = *V*_*reset*_	
**D**		**Input**	
**Type**		**Description**	
Poisson generator		Fixed rate, *N*_*ext*_ poisson generators per neuron, each one projects to one neuron	

**Table 2:** Parameters used in the simulations.

Parameter	Value	Parameter	Value
*C*_*m*_ (excitatory)	0.5 nF	*V*_*E*_	0 mV
*C*_*m*_ (inhibitory)	0.2 nF	*V*_*I*_	−70 mV
*f*	0.2	*V*_*L*_	−70 mV
*g*_*AMPA*,*ext*_ (excitatory)	2.08 nS	*V*_reset_	−55 mV
*g*_*AMPA*,*ext*_ (inhibitory)	1.62 nS	*V*_*θ*_	−50 mV
*g*_*AMPA*,*rec*_ (excitatory)	0.104 nS	*w*_+_	1.500
*g*_*AMPA*,*rec*_ (inhibitory)	0.081 nS	*w*_−_	0.878
*g*_*GABA*_ (excitatory)	1.287 nS	*α*	0.5 ms^−1^
*g*_*GABA*_ (inhibitory)	1.002 nS	*λ*_ext_	2.4 kHz
*g*_*NMDA*_ (excitatory)	0.327 nS	*τ*_*AMPA*_	2 ms
*g*_*NMDA*_ (inhibitory)	0.258 nS	*τ*_*GABA*_	10 ms
*N*_*E*_	800	*τ*_*NMDA*,*decay*_	100 ms
*N*_*I*_	200	*τ*_*NMDA*,*rise*_	2 ms
*N*_ext_	800	Δ*λ*	[0 30] Hz
*λ*	[15–140] Hz		

We use a mean-field reduction [24] in order to study the space of the principal parameters of the network.

The mean-field approximation allows reduction of the number of dynamical variables, by describing the average firing rate of each neuronal pool in the limit of an infinitely large number of neurons. In our network several different stationary states are possible depending on model’s parameters: spontaneous state, where the selective pools have low activity, three selective states with one selective pool firing at a high level and the others inhibited, a mixed state with two stimulated pools highly active. With some parameter values the network could sustain just one stable state, or could show bistable, behaviour, or could show multistable behaviour in which the spontaneous state or the mixed state and each of the two decision states are all possible stable states when the inputs to the network are being applied [24].

Note that although the mean-field reduction is useful to explore the attractor’s landscape of the model, it would probably not be adequate to account for data in [3]: in that experiment both behaviour and neurons activity are affected by the duration of the stimulus. The mean-field reduction assumes that the network is in a stationary state and hence is not able to account for these dynamical effects. Clearly different models (with noisy dynamics) based on the mean-field approximation could be more adequate.

#### Mean-field approximation

Here we detail the mean-field approximation of our model [24]. Under this approximation the stationary dynamics of each population can be described by the *population transfer function*, which provides the average population rate as a function of the average input current. The set of stationary, firing rates for the different neuronal pools can be found by solving a set of coupled self-consistency equations.

The mean-field approximation assumes that the network of integrate-and-fire neurons is in a stationary state. In this formulation the potential of a neuron is calculated as:

$$\tau_{x}\frac{dV(t)}{dt}=-V\left(t\right)+\mu_{x}+\sigma_{x}\sqrt{\tau_{x}}\eta \left(t\right)$$

where *V*(*t*) is the membrane potential, *x* labels the populations, *τ*_*x*_ is the effective membrane time constant, *μ*_*x*_ is the mean value the membrane potential would have in the absence of spiking and fluctuations, *σ*_*x*_ measures the magnitude of the fluctuations and *η* is a Gaussian process with absolute exponentially decaying correlation function with time constant *τ*_*AMPA*_. The quantities *μ*_*x*_ and $\sigma_{x}^{2}$ are given by:

$$\mu_{x}=\frac{\left(T_{ext}\nu_{ext}+T_{AMPA}n_{x}^{AMPA}+\rho_{1}n_{x}^{NMDA}\right)V_{E}+\rho_{2}n_{x}^{NMDA}\left\langle V\right\rangle +T_{I}n_{x}^{GABA}V_{I}+V_{L}}{S_{x}}$$ (1)

$$\sigma_{x}^{2}=\frac{g_{AMPA,ext}^{2}{\left(\left\langle V\right\rangle -V_{E}\right)}^{2}N_{ext}\nu_{ext}\tau_{AMPA}^{2}\tau_{x}}{g_{m}^{2}\tau_{m}^{2}}.$$ (2)

where *ν*_*ext*_ is the external incoming spiking rate, *ν*_*I*_ is the spiking rate of the inhibitory population, *τ*_*m*_ = *C*_*m*_/*g*_*m*_ with the values for the excitatory or inhibitory neurons depending on the population considered and the other quantities are given by:

$$S_{x}=1+T_{ext}\nu_{ext}+T_{AMPA}n_{x}^{AMPA}+\left(\rho_{1}+\rho_{2}\right)n_{x}^{NMDA}+T_{I}n_{x}^{GABA}$$ (3)

$$\tau_{x}=\frac{C_{m}}{g_{m}S_{x}}$$ (4)

$$n_{x}^{AMPA}=\sum_{j=1}^{p}r_{j}w_{jx}^{AMPA}\nu_{j}$$ (5)

$$n_{x}^{NMDA}=\sum_{j=1}^{p}r_{j}w_{jx}^{NMDA}\psi \left(\nu_{j}\right)$$ (6)

$$n_{x}^{GABA}=\sum_{j=1}^{p}r_{j}w_{jx}^{GABA}\nu_{j}$$ (7)

$$\psi \left(\nu \right)=\frac{\nu \tau_{NMDA}}{1+\nu \tau_{NMDA}}\left(1+\frac{1}{1+\nu \tau_{NMDA}}\sum_{n=1}^{\infty }\frac{{\left(-\alpha \tau_{NMDA,rise}\right)}^{n}T_{n}\left(\nu \right)}{(n+1)!}\right)$$ (8)

$$T_{n}\left(\nu \right)=\overset{n}{\underset{k=0}{\sum }}{\left(-1\right)}^{k}\left(\begin{array}{c} n\\ k \end{array}\right)\frac{\tau_{NMDA,rise}\left(1+\nu \tau_{NMDA}\right)}{\tau_{NMDA,rise}\left(1+\nu \tau_{NMDA}\right)+k\tau_{NMDA,decay}}$$ (9)

$$\tau_{NMDA}=\alpha \tau_{NMDA,rise}\tau_{NMDA,decay}$$ (10)

$$T_{ext}=\frac{g_{AMPA,ext}\tau_{AMPA}}{g_{m}}$$ (11)

$$T_{AMPA}=\frac{g_{AMPA,rec}N_{E}\tau_{AMPA}}{g_{m}}$$ (12)

$$\rho_{1}=\frac{g_{NMDA}N_{E}}{g_{m}J}$$ (13)

$$\rho_{2}=\beta \frac{g_{NMDA}N_{E}\left(\left\langle V_{x}\right\rangle -V_{E}\right)\left(J-1\right)}{g_{m}J^{2}}$$ (14)

$$J=1+\gamma \exp(-\beta \left\langle V_{x}\right\rangle )$$ (15)

$$T_{I}=\frac{g_{GABA}N_{I}\tau_{GABA}}{g_{m}}$$ (16)

$$\left\langle V_{x}\right\rangle =\mu_{x}-\left(V_{thr}-V_{reset}\right)\nu_{x}\tau_{x},$$ (17)

where *p* is the number of excitatory populations, *r*_*x*_ is the fraction of neurons in the excitatory *x* population, *ω*_*j*,*x*_ the weight of the connections from population *x* to population *j*, *ν*_*x*_ is the spiking rate of the *x* excitatory population.

The spiking rate of a population as a function of the defined quantities is then given by:

$$\nu_{x}=\phi \left(\mu_{x},\sigma_{x}\right),$$ (18)

where *ϕ* is the transduction function of population *x*, which gives the output rate of a population *x* in terms of the inputs, which in turn depend on the rates of all of the populations.

$$\phi \left(\mu_{x},\sigma_{x}\right)={\left(\tau_{rp}+\tau_{x}\int_{\beta (\mu_{x},\sigma_{x})}^{\alpha (\mu_{x},\sigma_{x})}du\sqrt{\pi }\exp\left(u^{2}\right)\left[1+\text{erf}\left(u\right)\right]\right)}^{-1}$$ (19)

$$\alpha \left(\mu_{x},\sigma_{x}\right)=\frac{\left(V_{thr}-\mu_{x}\right)}{\sigma_{x}}\left(1+0.5\frac{\tau_{AMPA}}{\tau_{x}}\right)+1.03\sqrt{\frac{\tau_{AMPA}}{\tau_{x}}}-0.5\frac{\tau_{AMPA}}{\tau_{x}}$$ (20)

$$\beta \left(\mu_{x},\sigma_{x}\right)=\frac{\left(V_{reset}-\mu_{x}\right)}{\sigma_{x}}$$ (21)

with erf(*u*) the error function and *τ*_*rp*_ the refractory period which is considered to be 2 ms for excitatory neurons and 1 ms for inhibitory neurons. To solve the equations defined by Eq (18) for all *x*s we integrate numerically Eq (17) and the differential equation below, which has fixed point solutions corresponding to Eq 18:

$$\tau_{x}\frac{d\nu_{x}}{dt}=-\nu_{x}+\phi \left(\mu_{x},\sigma_{x}\right).$$ (22)

For the numerical integration we used an Euler routine with a step size of 0.1 ms.

We also calculate the boundary of the attraction basins of fixed points (shown in Fig 3). In order to find the boundaries of one attractor we set the initial values of each pool to the firing rate of that pool in the attractor, then we varied the firing rate of pools L and R over different points in the plane *ν*_*L*_, *ν*_*R*_ and record which attractor the network falls in; we change the initial values of L and R with a bisection algorithm until finding the boundary with a precision of 0.1 Hz.

#### Parameters tuning

We adjusted the parameter Δ*λ* in order to obtain a qualitative fit of the probability of a correct response in trials without “sure target”. We then adjusted the target input to pool S in order to obtain proportions of “sure” choices comparable to those of the monkeys. The set of parameters used in the simulation is summarized in Table 2.

#### Stimulation protocol

**Fig 2:**
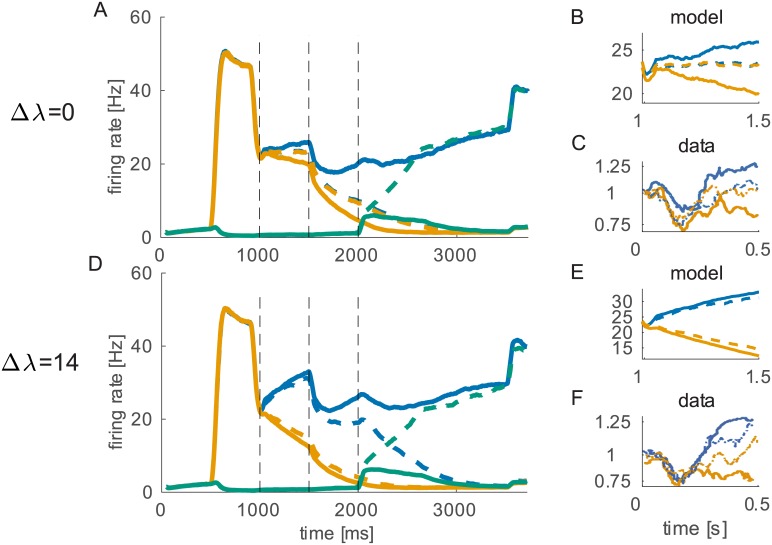
Neurophysiological activity of the model. Orange/blue/green lines represent R/L/S pool firing rates. Continuous lines show average firing rates of trials when either L or R was chosen. Dashed lines are average rates of trials when the sure option was chosen (i.e. pool S won). The network shows different dynamics depending on the winning pool. When the sure target was chosen, the firing rates of the L and R pools remain similar during input presentation. This behavior closely resembles the one observed in LIP by [3]. A: Average firing rates for Δ*λ* = 0. B,E: magnification during stimulus period. C,F: experimental data (showing normalized firing rate), time is relative to motion input onset. D: Average firing-rates for Δ*λ* = 14 Hz (correct trials).

The stimulation protocol was designed to match the input to LIP and is shown in Fig 2A. On each trial, after a first brief spontaneous period (500 ms), pools L and R receives an input due to the targets. After 500 ms the motion input is delivered to R and L. LIP neurons receive motion information from middle temporal area neurons encoding motion. The response of middle temporal neurons is almost linearly related to the motion coherence [19]. Consequently we increased the difference between inputs to population R and population L, according to the prevalent direction of dots motion. After motion stimulus (between 100–500 ms) the input to the pools is reset to the spontaneous values (3 Hz), to model the blank screen of the delay period (until the end of the trial). In forced choice trials, i.e. when the sure target is not presented, pool S receives no input but the spontaneous activity (3 Hz). In free choice trials, 500 ms after the extinction of the motion input, pool S receives an input that model the presentation of the sure target. Recently it has been found that the firing rate of LIP neurons during target presentation is proportional to the reward associated with the target [42], therefore we set the target related input to a higher value for high stakes pools (R,L) respect to the “sure” pool (S). Pool S does not receive inputs from the motion stimulus since the at the time of stimulus presentation no “sure” option target is present on the screen and the subject doesn’t know whether it will be shown later or not. An alternative approach could be to set the input to S to be dependent on the coherence of the RDM stimulus. This would increase the freedom of the system and the model would probably reach even better fit of experimental results. However we used the simpler approach (constant input) since we wanted to prove that the decision network *per se*, even with very simple inputs, is capable to reproduce the results of the uncertain option task.

### Probability of “sure” target selection: P(*S*|*ν*_*L*_, *ν*_*R*_)

We calculate the probability of choosing the “sure” option conditioned on the value of firing rate of decision populations R and L. In order to calculate this probability we divided the plane *ν*_*L*_,*ν*_*R*_ in square bins of side 1 Hz. We calculated for each trial the time averaged firing rate of decision pools in the 50 ms time window preceding the “sure” target onset. Then each bin in the plane is associated with those trials presenting the average firing rate before “sure” target onset determined by the position of the bin. Finally we counted for each bin how many trials ended up in a “sure” decision and divided by the total number of trials associated with that bin. We did this analysis separately for each stimulus duration collapsing together all coherences (Δ*λ*) and also for each coherence collapsing together all durations. The results were very similar across different conditions therefore we decided to pool together all trials from all stimulus conditions in order to show cleaner results.

### Decision time

We calculate the decision time of the network as the time when the firing rate of a decision pool crosses a threshold, *ϑ* set at 28 Hz. In addition the firing rate needs to be above the threshold in the subsequent 50 ms. Of course this values are not meaningful and other similar values give the same qualitative results. The reaction time is the sum of the DT and a non-decision time that is usually used to account for sensory processing and motor planning. Since we are not comparing the decision times of the model with experimental data we are not considering any non-decision time.

### Reward amount

We calculate the reward amount received by the subjects as a function of the probability of correct response and the probability of “sure” target selection. We arbitrarily consider the value of a correct response to be 1 and that of an error to be 0. The value of the “sure” target has been set to 0.8, since in the experimental setup the subjects received for a “sure” response approximately 80% of the reward associated to the high stakes targets. However we note that this value neither is crucial for our results nor need to be fixed based solely on the experimental setup. Indeed, while the value attributed by the subject to the “sure” option will reflect the objective amount of reward associated, it won’t probably be the same since it will include subjective preferences like risk aversion or risk attraction. We calculated the total reward in a block of trials as the sum of the reward received in each trial according the choice made (error: 0; correct: 1; sure: 0.8). Reward functions can be complicated and include many variables like reaction times, penalty time associated to errors, etc. [43]. We decided to use a very simple function since each of these variables need to be weighted based on the subjective value assigned to it, as for the value of the “sure” option, and we don’t have information about these subjective values.

### Distribution of number of error trials

For each combination of values of Δ*λ* and stimulus duration, we separated the trials in fast and slow using the minimum of DT histogram in the range [1001500] ms as the separation point. The restriction on the range of values of DT is necessary to find the dip in the distribution and not an eventual very small tail. We constructed the distribution of the number of error trials in fast and slow trials using bootstrap technique with 10000 samples. We then performed a t-test on these distributions in order to compare their means. The p-value of all tests was smaller than numeric resolution.

## Results

### A simple decision mechanism accounts for the uncertain option task

#### Experimental task and main results

The typical sequence of events in the uncertain option task employed in [3] is represented in Fig 1A. The subject is required to maintain fixation in the center of the screen in order to start a trial, then the targets appear on two opposite positions corresponding to the two possible decisions about the direction of motion. The motion stimulus is then showed for a variable duration followed by a variable delay period during which the monkey has to hold the decision in memory. The end of the delay period is marked by a go signal that triggers the saccade of the subject towards the decided motion direction. In a random half of the trials, during the delay period, the monkey was presented a third “sure” target. The election of this target corresponds to waiving the decision but was always rewarded with a smaller amount of liquid than the direction targets. The proportion of trials on which the decision was waived is considered by the authors as a measure of average decision confidence in a block of trials. They found that the probability of choosing the “sure” target (P(*S*)) decreases both for longer stimulus durations and for higher motion coherences (which should correspond to more certain conditions, Fig 1D upper row) and may therefore be considered as a proxy for confidence. They also found that the probability of a correct choice, when the “sure” target was shown but waived was higher than in trials without “sure” target presentation. This finding is thought to be crucial to prove the metacognitive nature of the task. Indeed, given the correlation of confidence and probability of correct responses, one way to improve performance would be to decline trials with low confidence (the same argument was already used in [17]). However this is not the only way to improve performance in this task: If subjects are associating the third option with an intermediate category [18], e.g. “flickering stimulus” in the case of the random dot motion (RDM) stimulus, they could also increase the probability of correct responses. Indeed when there is less apparent motion and the stimulus appears more flickering, the decision will be very difficult and will be associated with a low probability of being correct. Declining on these trials would then improve the performance of the subject.

#### A multiple choice decision model

Here we describe a multiple choice decision model implemented in a biologically detailed network. We will show that such a network is able to reproduce the experimental results [3] in accordance with the “intermediate category” hypothesis introduced above. Fig 1C graphically summarizes network details. The network has one population of neurons for each possible choice: Population R and L, for right and left targets, and population S for the “sure” target. Connections strength was designed in order to implement a competition mechanism between pools [19]. This type of model has been used to represents a decision making network of neurons in the area LIP both in binary [19] and multiple choice scenarios [16]. Pools L and R receive a stimulus dependent input (*λ*_*L*_ and *λ*_*R*_ respectively) that triggers the competition. During the trial one of the two pools will prevail on the other and enter into a decision state (high firing rate). Unbalanced inputs (Δ*λ* = *λ*_*L*_ − *λ*_*R*_ > 0) can bias the decision, but given the intrinsic stochasticity of the dynamics, an incorrect decision may be taken even when input currents are very different. Our model implements a simple multiple choices decision-making mechanism [16]: if three synchronous inputs are given to the network it would just take a decision about which of the three inputs is the largest. In order to mimic the experimental protocol [3], we used an asynchronous stimulation protocol, where the pools R and L are stimulated first and pool S is stimulated later. The first stimulation produces a competition between L and R and only after receiving the external input pool S enters the competition (see Fig 1B).

As shown in Fig 1D (bottom row) our model is able to reproduce qualitatively the behaviour of subjects in the experiment [3]. Note that the proportions of correct responses when the “sure target” was shown but waived, are very similar to those recorded experimentally [3], in particular they are higher compared to forced choice trials, i.e. when the sure target is not presented. We remark that the parameters were not adjusted to reproduce this feature.

In addition to psychophysics our model also reproduces the main neurophysiological results. We note that also for the neural activity no further tuning of the parameters was necessary to reproduce experimental data. Forced choice trials show the typical separation of firing rates when the chosen target was inside or outside the response field of the neuron [20–23]. Neurons in pool S on these trials have just baseline spontaneous firing activity (∼2 Hz). Firing rates of L and R pools on these trials are very similar to those in trials where the sure target was shown but waived. In this condition, as shown in Fig 2B and 2C (solid lines), after the targets presentation the model receives input from the RDM stimulus. This input triggers a competition between the two pools, L and R, soon one of them prevails, increasing the firing rate. During the delay period the firing rate of the winning pool remains high, keeping trace of the decision [24]. When the input to pool S is turned on, the high activity of the winning pool is inhibiting, through inhibitory neurons (see Fig 1), the other pools preventing population S neurons from increasing their firing rates independently. When the sure target is chosen (Fig 2B and 2C dashed lines), due to the intrinsic stochasticity of the system, the firing rates of L and R do not diverge as much as in trials where the sure target is waived. On these trials typically pool S receives less inhibition and therefore can increase the firing rate when stimulated by the presentation of the sure target and eventually win the competition against R and L.

The reduced separation of firing rates is a key characteristic of LIP neurophysiology in the uncertain option task [3]. The insets in Fig 2B and 2C show that our model can qualitatively reproduce the neurophysiological findings of [3].

### Analysis of network dynamics

**Fig 3:**
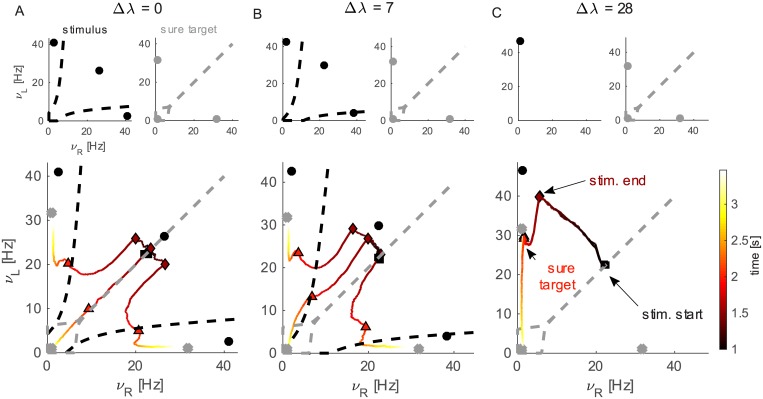
Attractors and their basins represented in the *ν*_*L*_, *ν*_*R*_ plane with mean dynamics. A, Top: attractors landscape for Δ*λ* = 0. Attractors (dots) and their basins (dashed lines) during stimulus condition and sure target condition. Bottom: Traces show average dynamics of correct, error and sure choices (depending on the final point reached by the trace). The color of the trace represents time. Relevant events during the trial are marked by symbols as explained in the figure. Attractors and basins as in top panels are shown for reference (dots of sure target condition replaced by crosses for clarity). B: Attractors landscape and dynamics as in A, for Δ*λ* = 7. C: Attractors landscape and dynamics as in A, for Δ*λ* = 28. Note that the traces corresponding to left and sure choices are overlapped but left trials remain near the gray decision memory attractor.

In order to get more insight into the dynamics of the system we can observe the evolution of the decision process in the phase plane of firing rates *ν*_*R*_ and *ν*_*L*_, as depicted in Fig 3. We can observe the attractor landscape and the dynamics of the model in this landscape: top panels show attractors and their basins, while bottom panels show the mean dynamics, as detailed below. In attractor neural network (ANN) models usually each choice is represented by one attractor. Whenever the system enters into the basin of attraction of a decision attractor it will remain there (unless stimulation changes; e.g. in the case of time varying stimulation the system can escape from decision boundary) and the corresponding decision will be taken [25, 26]. Bistable systems (where only two decision attractors are stable) are usually enough to solve binary decision tasks. Our hypothesis is that an additional non-decision attractor may be helpful in a delayed stimulation protocol. In essence, we exploited the multistable regime (where two decision attractors and a non-decision attractor are stable) to represent the uncertain option task.

Since pool S has constant activity around 2-3 Hz until the onset of the sure target, we disregard this pool for this analysis and only consider the projection of the system dynamics onto the *ν*_*R*_, *ν*_*L*_ plane that provides useful and clearer information about the decision process.

In Fig 3 we plot the attractors and their basins in the phase plane of the system (the mean-field reduction of the spiking network) for three different values of Δ*λ* (*λ* = 50 Hz, see sec. *Multistability enhances flexibility in delayed decisions* for an exploration of this parameter). We represent attractors and their basins under two different stimulation conditions that are given during a trial (Fig 3 top panels): stimulus and sure target. The delay condition, where *λ* = 0 Hz, is very similar to the sure target condition (the only difference is that *λ*_*S*_ = 0 Hz is the former and *λ*_*S*_ > 0 Hz in the latter), hence we omit it here to keep the figure uncluttered; the attractor landscape for the delay condition is shown in S1 Fig. Black circles mark the attractors position when the RDM stimulus is on and their basins boundaries are represented by dashed black curves. In Fig 3A and 3B, the region of the plane laying between the two boundaries is the basin of the “mixed” non-decision attractor (black dot on the diagonal). During the delay period the stimulus is off and the attractor landscape changes as depicted in S1 Fig, showing a resting state attractor and two decision memory attractors. When the sure target is shown a decision attractor appears corresponding to the sure target choice (top right panel for each value of Δ*λ* in Fig 3). In this attractor *ν*_*L*_ = *ν*_*R*_ ≈ 2 Hz, while *ν*_*S*_ >>0. Also decision attractors for left and right choices exist with similar symmetric conditions.

The traces in the bottom panels show the trial average dynamics of the spiking network for decisions left (correct), right (error) and sure target. The color of traces represents the evolution of time from black (corresponding to stimulus onset, marked by a black square), to yellow (the end of the trial), passing through red. Traces terminating in the L (R) basin correspond to left (right) choices. Traces terminating in the sure choice attractor basin (gray dashed lines) correspond to sure choices. Events of the trial are represented on the traces by different symbols (as indicated in panel C bottom). Attractors and their basins are depicted also in bottom panels for reference (attractors during sure target period are represented in bottom panels by gray crosses for clarity). When the motion stimulus is turned on, both populations present relatively high and comparable firing rate due to the previous response to the direction targets.

At Δ*λ* = 0 (Fig 3A) the attractors basins are symmetric and so are the mean dynamics: the system moves towards the corresponding decision attractor when the stimulus is on (between black square and brown diamond) but, due to the presence of the “mixed” attractor it doesn’t reach the decision attractor basin. When the stimulus is turned off (brown diamond) all three traces move diagonally towards the spontaneous state. Then the traces of left and right choices are attracted towards the respective decision attractors and when the sure target is turned on (orange triangle) they are already into the basin of attraction of the decision state. On the contrary the trace representing sure target choices keeps moving on the diagonal and when sure target appears the system is near to the sure attractor.

When Δ*λ* > 0 the attractor landscape becomes asymmetric. For intermediate values of Δ*λ* the behavior of the system is similar to that at Δ*λ* = 0 although slightly biased towards the left (correct) attractor (see Fig 3B). For higher values of Δ*λ* eventually the right and mixed attractors disappear and only one decision attractor exists during stimulus presentation. In this condition the system moves faster towards the decision state. Anyway, when stimulus is turned off, the firing rate of the winning pool decreases and eventually on some trials the sure target is chosen. Note that in panel C bottom the traces corresponding to left and sure choices are overlapped but left trials remain near the gray decision memory attractor while sure trials reach the attractor near the origin of the axes.

#### Predictions of rate distribution and its relation to sure choices

We want to show what happens in single trials and what are the conditions, which determine a sure choice. A natural hypothesis is that the proximity of the system to the basin of attraction of the sure target (green dashed line) drives the system to choose the sure target.

**Fig 4:**
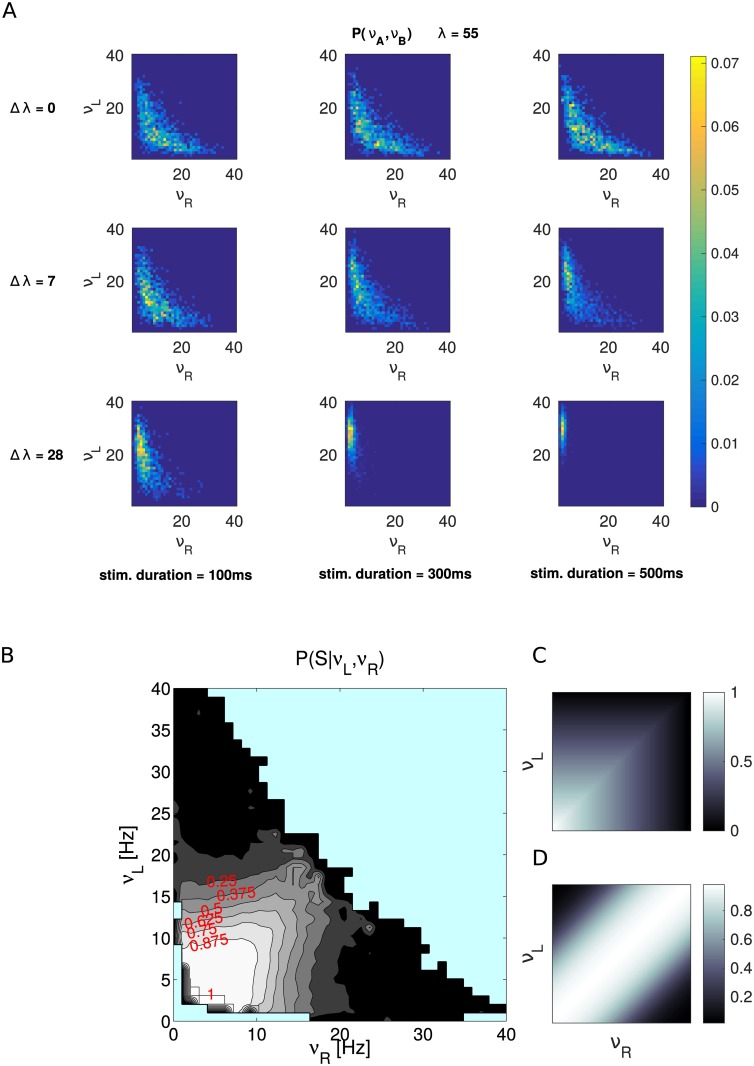
Probability distributions of firing rates and conditional probability of sure choices. We represent the state of the spiking network using the mean firing rate of pools L and R in the 50 ms before the “sure” target onset, *ν*_*L*_ and *ν*_*R*_ respectively. A: Each panel shows the probability distribution of *ν*_*L*_ and *ν*_*R*_ for a given combination of stimulus evidence (Δ*λ*) and stimulus duration (calculated using 1000 trials). B: Conditional probability of choosing the sure target given the state of the system in the *ν*_*L*_,*ν*_*R*_ plane. Light blue indicates points with less than 30 trials. C: Cartoon showing the idealized P(*S*|*ν*_*L*_, *ν*_*R*_) ≈ *max*{*ν*_*L*_, *ν*_*R*_} predicted by the model. D: Cartoon showing the corresponding prediction of the model proposed in [9]: P(*S*|*ν*_*L*_, *ν*_*R*_) ≈ *σ*(|*ν*_*L*_ − *ν*_*R*_|).

First, we represent the state of the system showing the probability distribution of firing rates of the full spiking network at the time of sure target onset (Fig 4A). Then we show the probability of an S choice given the state of the system (i.e. *ν*_*L*_ and *ν*_*R*_). These probability distributions are interesting predictions of our model that could be easily tested in an experimental set up, where the activity of decision neurons selective for the two choices is recoded simultaneously. Such an experiment would be highly valuable in order to falsify this model and to distinguish it from the model in [9], as explained at the end of this section.

The panels of Fig 4A represent different conditions: rows from top to bottom are distributions for increasing values of stimulus coherence, mapped by the variable Δ*λ*, while columns from left to right represent increasing durations of the stimulus. When there is very low evidence (or no evidence at all, like in the first row) the distribution of *ν*_*L*_, *ν*_*R*_ is quite symmetric about the diagonal and the duration of the stimulus has very low or no effect. In the second row we can observe that a moderate level of coherence already skews the distribution towards the correct decision area (points under the diagonal correspond to trials evolving towards the wrong attractor). When duration of the stimulus increases, the skewness gets larger. On the bottom row (very high stimulus coherence), for a short duration, the distribution appears markedly skewed towards the decision area and we can observe that for longer stimulus durations the distribution moves even further into the decision region concentrating towards the correct decision attractor.

We calculate now the probability of a sure response given the state of the system, i.e. the conditional probability P(*S*|*ν*_*L*_, *ν*_*R*_) as shown in Fig 4B. In accordance to our hypothesis we found that the probability of choosing the sure option decreases when moving away from the basin of S choice (see Fig 4B). In general the distribution of P(*S*|*ν*_*L*_, *ν*_*R*_) reproduces the shape of the attraction basin of the sure target as calculated with the mean-field approximation (green dashed lines in Fig 3).

Considering only the state of the system at the moment the sure target is shown is an approximation that neglects the previous history of the trial. However we note that P(*S*|*ν*_*L*_, *ν*_*R*_) does not change when the stimulus parameters (average intensity *λ*, duration and Δ*λ*) are changed (see S3 Fig). Different values of these parameters induce different dynamics of the system, for example for *λ* = 55 Hz the “mixed” attractor produces a slower decision dynamics that spends more time around the diagonal, while for *λ* = 15 Hz there is no “mixed” attractor and the dynamics is faster and more direct. Therefore, the fact that P(*S*|*ν*_*L*_, *ν*_*R*_) does not change with *λ* may indicate that the state of the system before sure target onset is enough to characterize the sure target choice behaviour and that the complete trajectory of the system is not necessary. The parameters of the stimulus (coherence, duration and *λ*) affect instead the distribution of firing rates in the *ν*_*L*_, *ν*_*R*_ plane (as summarized in Figs 4A and S2) and this in turn produces different probabilities of P(*S*).

Finally, we note that in the model the activity of the pool that is dominating the competition is important to determine the probability of a sure choice. Indeed we can observe that the contour lines in Fig 4B form approximately square surfaces similar to the basin of attraction of the sure target. This means that in general the network only takes into account the *max*{*ν*_*L*_, *ν*_*R*_}. This prediction is particularly relevant since different prediction is proposed in another model [9] of the same data (see in particular their Fig 6A and related text). The authors in that study [9] propose that 1 − P(*S*), a quantity that they call “confidence”, is a sigmoidal function of the absolute value of the difference between the activity of pool L and R. Similarly in our model 1 − P(*S*) as a function of the absolute value of the difference between the activity of L and R also resembles a sigmoid function (see S4A Fig). However in our model, also as a function of the summed activity of L and R, 1 − P(*S*) resembles a sigmoid function (see S4B Fig). This means that, although our model behaves similar to the continuous attractor model, in our model the activity of both decision pools are informative about the probability of a sure response. Since no other variable is mentioned as informative about the probability of a sure response [9] we can extrapolate their prediction in the *ν*_*L*_, *ν*_*R*_ plane. Fig 4C and 4D present a simplified illustration of the two predictions: respectively that of the model proposed here and that of the continuous attractor model [9].

Panel C shows (with synthetic data) *max*{*ν*_*L*_, *ν*_*R*_}. Panel D shows (with synthetic data) an extrapolation of the prediction in [9] on the *ν*_*L*_, *ν*_*R*_ plane: *σ*(|*ν*_*L*_ − *ν*_*R*_|), where *σ*(*a*) is the logistic sigmoid function. The different predictions highlight the diverse mechanisms underlying the continuous and the discrete model, proposed here. A new experiment with the same behavioral setting could test these predictions and falsify one of the two (or both) models, if the activity of neurons selective for right and left choices would be recorded simultaneously.

#### Dynamics of correct and error trials

The definition of correct and error choices in forced choice trials is straightforward. In free choice trials the asynchronous stimulation protocol we used induces first a competition between L and R, while S enters into the competition only after the onset of the sure target. Hence we can also separate trials in correct and error “early” choices, until we constrain the analysis to the period before the stimulation of S.

**Fig 5:**
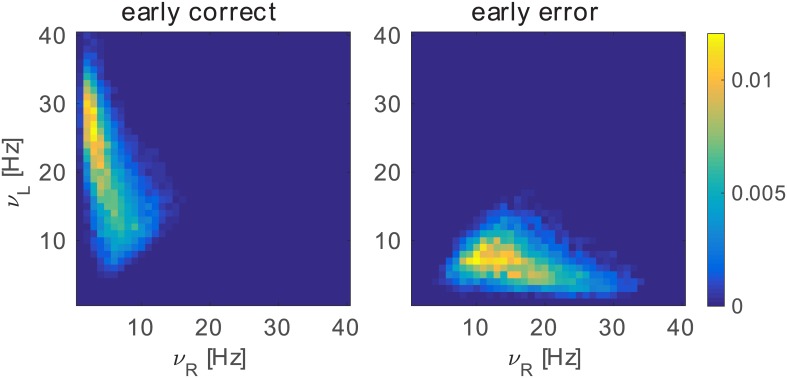
Probability distributions of *ν*_*L*_ and *ν*_*R*_ as in Fig 4 separated in early correct and error trials. Each panel shows the probability distribution of the state of the system at “sure” target onset for early correct (left) and early error trials (right) calculated using all stimulus durations and all values of Δ*λ* (29858 trials for early correct and 10142 trials for early error conditions).

Here we characterize the dynamics of the network, before sure target onset, separately for correct and error choices (Fig 5).

While early correct trials are distributed mainly in the decision region, early error trials are located near the diagonal. For example, in trials where the network dynamics is evolving towards the right choice but the evidence for the left choice is actually stronger, the system will remain longer near the basin of attraction of the sure choice. If the sure target is not presented (forced choice) these early error trials will likely end up in a wrong choice. However, if the sure target is presented (free choice), those early error trials which are near to the basin of attraction of the S choice will end up in sure choices. This configuration of the system explains the better performance of the model in free choice compared to forced choice trials.

### Multistability enhances flexibility in delayed decisions

In this section we study the modification of network attractor landscape induced by the input to decision pools L and R (*λ*). We will show that a key ingredient of the model for reproducing the data in [3] is the presence of three stable attractors (also known as multistable regime) during the stimulus phase. Usually decision-making tasks only require one attractor for each alternative [16, 19, 27]. An additional non-decision attractor, where decision pools have comparable firing rate, makes the decision process slower (eventually the system may remain in the undecided state). Therefore, we hypothesized that a non-decision attractor in the stimulus phase could bring more flexibility to the system in a delayed stimulation protocol, where the influence of options presented later has to be taken into account. We recall that during stimulus phase pool S only receives background input and is practically silent. Hence we consider only the activity of pools R and L.

**Fig 6:**
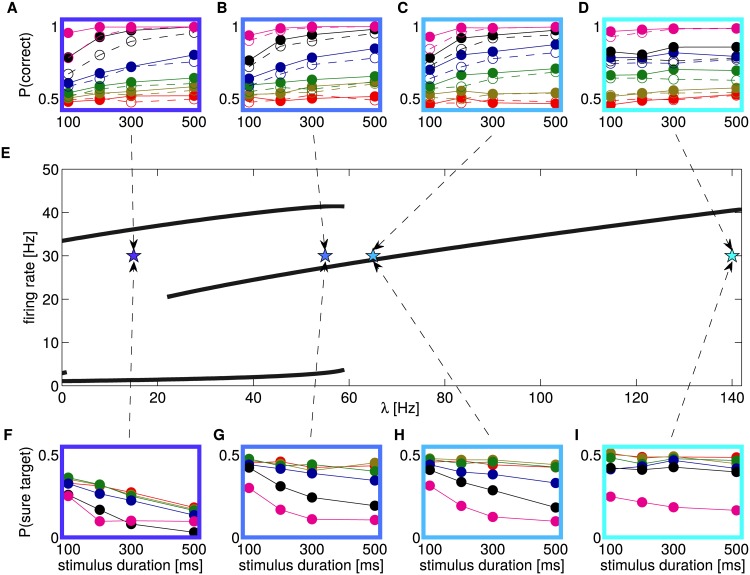
Bifurcation diagram and psychophysics measures in different regions. A-D: P(C) for trials when the sure target was (not) shown are represented with filled (open) circles. The color of the box and the arrows indicate the value of *λ* used. E: Bifurcation diagram over parameter *λ*. The curves represent the firing rates of pools L and R in the attractor (according to the mean-field reduction of the spiking network). Four regions appear (in order of increasing *λ*): A first tiny multistable region when both decision and spontaneous attractors appear; a bistable region where only the two decision attractor are present; a multistable region, where decision attractors coexist with a “mixed” attractor (both pool have intermediate firing rate); a monostable region, where only the “mixed” attractor exists. F-I: P(S) for different values of *λ* (color convention as in A-D). Error bars indicating the standard deviation have been omitted since they are always smaller than symbols.

Here we set the differential input Δ*λ* = 0 so that the system is symmetric regarding the two choices R and L. We show in Fig 6E the bifurcation diagram of the system (a mean-field equivalent of the spiking network) for parameter *λ*. This diagram shows, for each attractor, the firing rate of pools R and L as a function of *λ*, when the system converged to the attractor (for details see Methods).

As can be observed the variation of *λ* induces different dynamical regimes of the model. At *λ* = 0 Hz there is a “spontaneous attractor”, where both pools are in a spontaneous quiescent state around 3 Hz, and two decision attractors, where one pool is silent and the other fires at a high frequency. These decision attractors in absence of input are key for keeping in memory the decision during the delay period. For values of *λ* larger than 1 Hz the spontaneous attractor destabilizes and only the decision attractors remain stable. At *λ* = 21 Hz another bifurcation occurs and a stable non-decision or “mixed” state appears. This multistable region extends until *λ* = 59 Hz, where the decision attractors disappear and only the “mixed” state remains stable.

Panels A-D show the accuracy, P(*correct*), for 4 sample points in different regions of the bifurcation diagram (marked by stars). For each point we adjusted the parameter Δ*λ* in order to get a qualitative match with experimentally measured P(*correct*). Panels F-I show the P(*S*) for the same points. As can be seen the model is able to reproduce qualitatively the performance of monkeys both in the bistable and in the multistable region. Right after the pitchfork bifurcation, in the region where only the “mixed” state attractor is stable, the model still reproduce quite well the P(*correct*), while, if we further increase *λ*, moving away from the bifurcation, it is not possible to approximate the experimentally observed P(*correct*) since the model becomes insensitive to stimulus duration. Interestingly the P(*S*) changes qualitatively when moving through the different regimes of the network. In the bistable region (dark blue, panel F) the P(*S*) is less sensitive to changes in stimulus coherence (i.e. Δ*λ*), e.g. the frequency of S choices rapidly decreases as a function of stimulus duration for low evidence trials (red curve), while experimental data show a very flat curve. In addition, when the network is stimulated for 500 ms, the P(*S*) for different values of Δ*λ* show quite small variation, which is also inconsistent with experimental data. When moving towards the pitchfork bifurcation these effects are gradually reduced and a good qualitative fit of experimental data can be observed in the multistable region or right after the bifurcation.

This change of behavior can be understood considering the dynamics of the system in the phase plane and the conditional probability of a sure choice. We observe that in the bistable regime (*λ* = 15), for Δ*λ* = 0, the duration of the stimulus induces a strong change in the distribution of firing rates in the phase plane (S2 Fig): system state is spread over the diagonal and decision regions for short durations and centred on the two decision regions for long durations. Since the P(*S*|*ν*_*L*_, *ν*_*R*_) is very different in these two regions (see Fig 4B), the sure choices are strongly influenced by the duration of the stimulus. At the same time the distribution of firing rates for different values of Δ*λ*, when the network is stimulated for 500 ms, is very similar. On the contrary, in the multistable regime the distribution of *ν*_*L*_, *ν*_*R*_ (Fig 4A), for Δ*λ* = 0 or very small, show only little variation as a function of stimulus duration (compare with S2 Fig). As a consequence the sure choices for small values of Δ*λ* are not affected by stimulus duration. In S4 Fig we show an alternative representation of data fitting: The data of the model are represented as a scatter plot against the experimental data for different values of *λ*. In order to help the reader we also show the Pearson correlation coefficient between model and data. The values of *λ* near the bifurcation produce the best fit.

Interestingly, in all regimes the P(*C*) increases in trials when the “sure” target was shown but not chosen compared to trials without “sure” target (filled circles compared to empty circles in Figs 1D and 6A–6D). This effect does not depend on the dynamical regime of the network and is produced by the fact that the probability of an S choice is higher in trials that are more difficult due to random fluctuations of the input, as explained in sec. *Dynamics of correct and error trials*.

### Sure choices in correct and error trials

**Fig 7:**
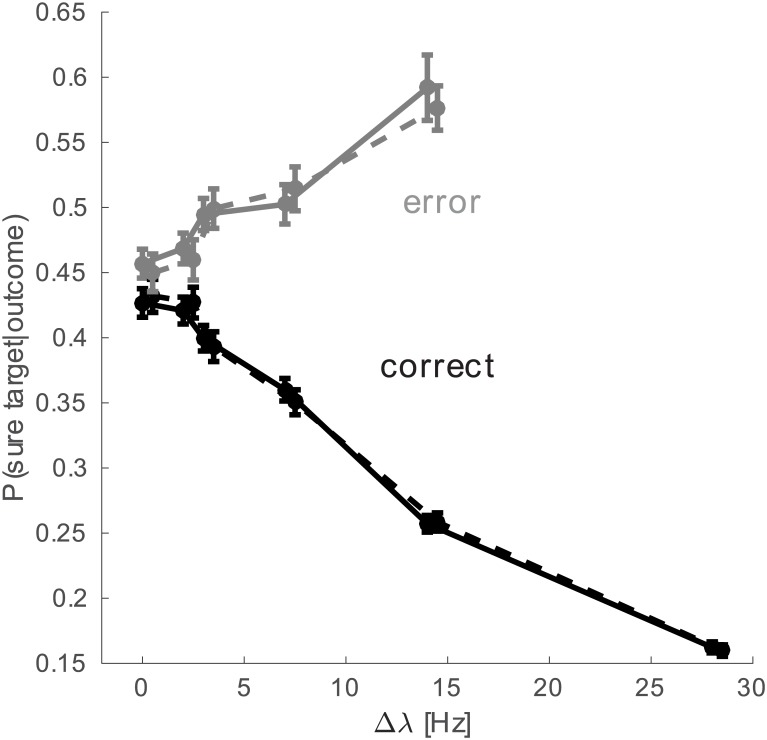
Probability of choosing the “sure target” as a function of Δ*λ* for early correct and error trials. Continuous line is the prediction from the spiking network. Dashed lines are predictions of the reduced probabilistic model (points are slightly shifted on the horizontal axis for better visibility).

In this section and in the following we provide two further predictions that may be tested in future experiments. The proportion of early error trials is expected to depend on the discriminability of the stimulus. This fact together with the distribution of firing rates in correct and error trials (see sec. *Dynamics of correct and error trials*) bring us to hypothesize that the P(*S*) should show an inverted pattern in early correct and early error trials, when plotted as a function of the discriminability (Δ*λ*). In Fig 7 we can see that the model predicts a decreasing proportion of “sure” choices in correct trials when the task gets easier but an increasing proportion of “sure” choices in error trials.

This pattern, is analogous to the X-shaped pattern usually associated with confidence [1, 28, 29] (indeed the model would give the same results for negative values of Δ*λ* and the pattern would be X-shaped).

This prediction arises by applying a decision criterion (a simple activity threshold crossing, see Methods) to the dynamics of the spiking model. However we can get a very similar prediction using a simple probabilistic model. Here the predicted quantity is the conditional probability of a sure choice given the outcome of the decision in the early stage of the trial, P(*S*|*outcome*). This quantity is unavailable if we look at the psychophysical measurements. The available measures are the probability of correct choices when the sure target was shown but waived, $\text{P}(C|\bar{S})$ (this is indeed shown in filled circles in Fig 6A–6D)i, and the probability of choosing the sure target, P(*S*). Using Bayes theorem and the fact that $P\left(S|C\right)+P\left(\bar{S}|C\right)=1$, we can write the probability of a correct response when the sure target was not chosen as a function of the probability of correct responses, the probability of choosing the sure target and the conditional probability of choosing the sure target given an early correct response:

$$P\left(C|\bar{S}\right)=\frac{P(C)-P(S|C)P(C)}{1-P(S)}.$$

Since all other quantities are known we can calculate

$$P\left(S|C\right)=\frac{P\left(C\right)-P\left(C|\bar{S}\right)+P\left(S\right)P\left(C|\bar{S}\right)}{P(C)}.$$

Using the same approach we can calculate the conditional probability of a sure choice given an early error as

$$P\left(S|E\right)=1-\frac{P\left(E|\bar{S}\right)P\left(\bar{S}\right)}{P(E)}.$$

This model can be considered as a simple probabilistic interpretation of our biophysically realistic model. The only assumption of this model is that the presentation of the sure target in the trial doesn’t change the overall probability of correct. The prediction given by this simple model is very close to that calculated with the spiking network and is plotted with dashed lines in Fig 7. Since this model is based solely on behavioral measurable quantities this prediction could be easily tested experimentally in order to verify or falsify the model.

The predicted X pattern is very relevant beyond the opportunity of falsifying our model. Indeed this pattern has been often associated with decision confidence [1, 28–30]. Nonetheless we note that cases of human confidence rating resulting in different patterns have already been reported [31–33]. Here we show that a multiple choice decision-making model also predicts the X-pattern, thereby providing further evidence of the disentangling of confidence and X-pattern.

### Multistable dynamics induces bimodal decision times

We describe next the decision times distributions and their relationship to the network attractor landscape. What we refer to as decision time (DT) is the time (from stimulus onset) that the model takes to integrate the stimulus signal and reach a decision. This time offers an insight into the decision process and could be compared to empirical reaction times of a modified version of this task. We consider here that a decision has been reached when the firing rate of one decision selective pool crosses a threshold, *ϑ* = 28 Hz for *λ* = 50 Hz. We chose this value for the threshold in order to have less than 5% of undecided trials when the go cue is shown (end of the trial). For other values of *λ* the value of the threshold is different but was always chosen according to the same criterion. Independently on the decision rule another problem remains still open when facing with delayed response tasks. Indeed if after accomplishing the decision rule but before the “go” signal the accumulation process keeps on, a change of mind [34] could occur. It is not clear whether the choice is based on the winning accumulator when the decision criterion is met (involving a separate working memory process) or based on the winning accumulator when the “go” signal is presented. We have considered that what matters is the winner when the threshold is crossed. In this scenario we report that in a 10 ± 2% of trials a change of mind, as described in [35], could occur. This amount of changes does not affect our results (results are qualitatively similar in the two situations).

**Fig 8:**
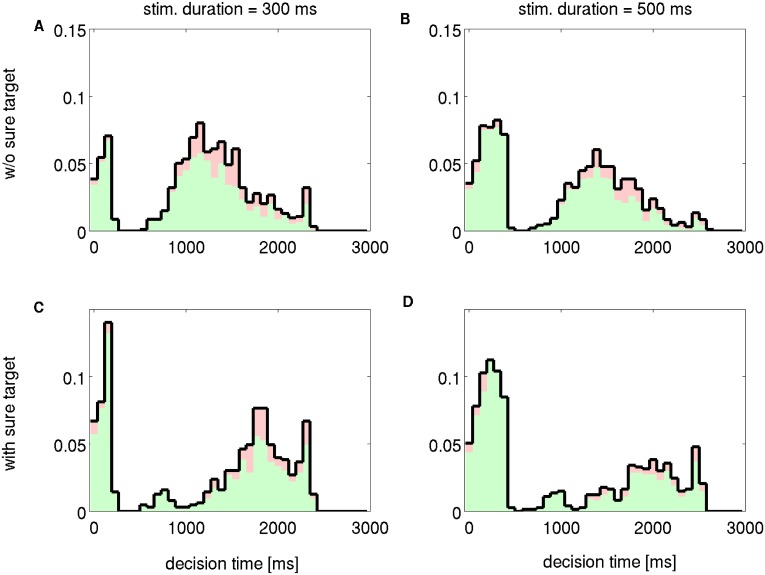
Decision times distributions for two different durations and in forced choice trials (upper row) and free choice trials (bottom row); Δ*λ* = 7 Hz for all panels. The color of the histogram represents the relative contribution of correct (green) and error (red) trials to the global distribution.

We observe that in forced choice trials, the DTs have a bimodal distribution. Fig 8A and 8B shows the DT probability distribution in trials when the “sure” target was not presented respectively for a short (300 ms) and a long (500 ms) duration of the stimulus. The distributions can be easily separated in a fast and a slow part. This bimodal distribution is verified for almost every value of the coherence. Indeed, when the coherence increases a larger proportion of trials fall in the fast part of the distribution. At some point, when evidence for the decision is very high, the fast and slow parts collapse into a unimodal left skewed distribution.

We note that the two parts of the distributions correspond to two different types of trials. On some trials the network reaches the decision criterion during the stimulus period and these trials result in fast responses. On the contrary when no decision is met during the stimulus period, once the stimulus is extinguished, the decision process relies only on the memory trace of the stimulus (i.e. the self-sustained activity of the network). These type of decisions are of course slower. We can observe that the fast part of the distribution comprises DT values that depend on the duration of the stimulus: in Fig 8A the fast part includes DTs of up to about 0.3 s, while in Fig 8B the fast group includes DTs of up to about 0.5 s.

The bimodality of these distributions is induced by the presence of the third “mixed” attractor. This stable point attracts the dynamics of the network during the stimulus driven regime. This happens on trials when the stimulus input is not strong enough to push the system into a decision basin. When the system is in the basin of attraction of the “mixed” state it will remain there until the stimulus extinguishes destabilizing the attractive point; in this case only after stimulus offset the network is eventually going to enter into the basin of attraction of one decision. As a confirmation we observe that when the mixed attractor is not stable, for example in the bistable regime, the DTs distributions are not bimodal (e.g. *λ* = 15Hz, S5 Fig). We note that the mechanism that induces bimodal DTs distributions is independent of sure target input, indeed also forced choice trials, i.e. without sure target, present such a bimodal distribution.

We separate the trials in correct and error; in Fig 8 the color of the histogram indicates the contribution of correct and error trials to the global distribution (green: correct; red: error). Errors occur predominantly in slower trials (p-value for t-test of bootstrap samples smaller than numeric resolution; see S8 Fig and Methods for details).

Coherently with the analysis presented so far, we found that the sure target is chosen more likely in slower trials. Indeed when the sure target is presented (Fig 8C and 8D), the slow part of the distribution has a smaller mass compared to the distribution of forced choice trials. Note that the apparent increased mass of early decisions is only a side effect of less slower decisions: since many slower trials end up in the sure target attractor they are not computed to build the distribution; consequently the total number of trials decreases compared to forced choice trials and the early decisions appear more frequently. The slower trials remain longer near the basin of attraction of the sure target and so the sure option will be chosen more frequently. This finding may indicate that a very large sure target input may drastically reduce the slower part of the distribution, thereby producing a unimodal skewed distribution. However such strong sure target input would also produce a very large amount of sure target choices overall, which would be a very different condition than the considered experiment [3].

The “mixed” attractor is not the only necessary ingredient to produce bimodal DTs distributions. Indeed it is also needed that the network reaches the decision threshold during stimulus period only in a subset of trials. Different parameters regulate the speed of the network and the study of these parameters is beyond the scope of this work. However substantial effects on DTs distributions can also be obtained by changing the decision threshold, *ϑ*. When *ϑ* is low enough that the decision is always taken during stimulus period, DTs have a left skewed distribution (see S6 and S7 Figs). At the other extreme, when *ϑ* is high enough that the decision is always taken after stimulus offset, DT distribution is right skewed. For intermediate values of decision threshold the DTs have a bimodal distribution (S6 and S7 Figs).

### Attractors landscape manipulates the flexibility of decision-making

We have shown that the current model is able to reproduce the experimental findings in [3] in the multistable regime, near the pitchfork bifurcation that separates the multistable from the monostable regime (see Fig 6). This is an unconventional result since ANNs designed for decision-making usually exploit the bistable regime [16, 19, 27]. Nonetheless in the present experimental paradigm a more complex dynamics could be beneficial. Indeed, in this scenario the subject needs to find a trade-off between accumulating evidence for each of the first two alternatives and remaining in a flexible state where the third alternative, when offered, can be chosen.

Here we analyze the behavior of the model for different values of parameter *λ*, the common input to the decision pools. This parameter can embrace many complex phenomena (e.g. stimulus contrast, attention, urgency, etc.), that we didn’t modeled explicitly since they seem to take place outside of the decision-making network. This analysis is interesting *per se* to understand the model. Moreover this analysis can supply predictions about novel experiments. In fact, it is believed that the subjects have the ability to change, to some extent, their *λ* in order, for example, to satisfy different sets of experimental instructions (e.g. “fast” *versus* “accurate” trials [36, 37]).

In contrast to previous analysis summarized in Fig 6, here we keep the values of Δ*λ* fixed when varying the value of *λ*. This will simulate the scenario of a subject changing its *λ* due to experimental manipulation, like time pressure, stress on accuracy, stimulus visibility, etc. In this scenario Δ*λ* shouldn’t be adjusted for each value of *λ* since, once the subject has learned the task, so that the performance is stable, Δ*λ* should only depend on stimulus coherence if all other experimental conditions are constant.

**Fig 9:**
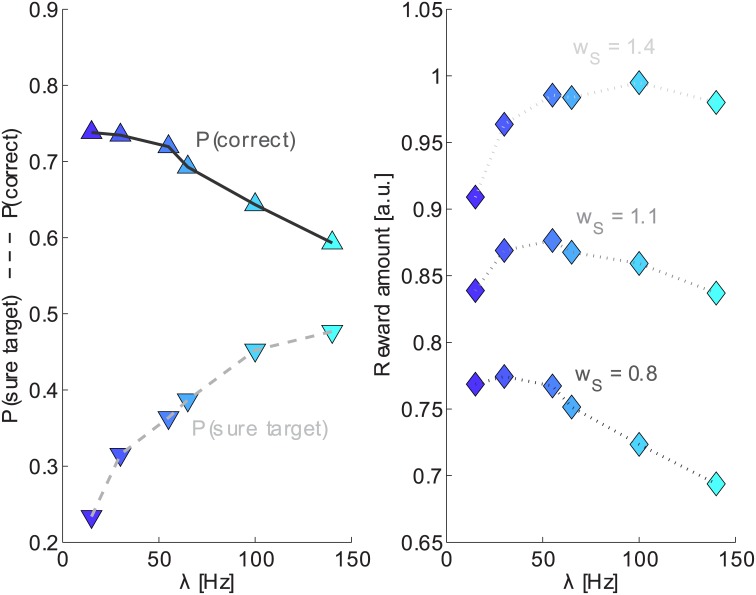
Trade-off between accuracy and flexibility. A: P(C) and P(S) as a function of *λ* (color as in Fig 6). B: Mean reward amount (P(*correct*) + P(*suretarget*) ⋅ *w*_*S*_) as a function of *λ*, for different values of *w*_*S*_.

Fig 9A shows the overall accuracy and P(S) for different values of *λ*. As expected the P(C) decreases when *λ* increases since the signal-to-noise ratio decreases. At the same time the P(S) increases as a function of *λ*.

This means that the subjects have to trade accuracy for flexibility. If the subjects want to make more correct choices they will consider less the third option presented later in the task. Conversely, if the subjects want to remain more flexible to future possible options they have to accept a higher error rate. Therefore we can infer how much subjects valued flexibility in this task. We can hypothesize that subjects were maximizing the mean reward amount. We calculated the mean reward amount over all trials using the equation: P(*correct*) + P(*S*) ⋅ *w*_*S*_, where *w*_*S*_ is the value of the third option. This is a simplified equation for the reward: we are not interested in the actual value of the third option but in its value relative to that of correct choices, so we fix the value of the correct option to one (the units of reward are then arbitrary). Fig 9B shows the reward amount as a function of *λ* and for different values of *w*_*S*_. The bottom curve shows the reward for *w*_*S*_ = 0.8, which is close to the relative amount of liquid reward used in [3] for the sure target choice. This curve is almost flat in the range 15–50 Hz of *λ*. The middle curve correspond to *w*_*S*_ = 1.1 and we can observe that in this case the reward amount increases until 50 Hz and then decreases again. For higher values of *w*_*S*_ (upper curve) the reward amount keeps increasing (or remains high) over the entire range of *λ*. Consequently, a prediction of the model is that the relative value of the sure target is not simply proportional to the amount of liquid associated to the sure option, rather subjects would assign a higher value to the sure target. This prediction is based on three hypothesis: 1) The value of *λ* used by the subjects in the experiment is the one that best fit the experimental data, i.e. multistable region *λ* ≈ 55 Hz. 2) The subjects have the ability to internally control *λ* in response to some experimental change. 3) The subjects are trying to maximize the proposed mean reward amount. If we consider these hypothesis plausible, than we may say that subjects were assigning a large value to the sure target in order to be more flexible and to remain open to new options offered at a later stage in the trial. However we note that subjects, although flexible, were far from optimality (in terms of liquid reward). For example, they were choosing the sure option only 60% of the trial when their performance was at chance.

## Discussion

We presented a biologically detailed network model that can account for behavioral and neurophysiological measurements related to decision confidence. We characterized the dynamics of the model in terms of the underlying attractor landscape and showed how the landscape is related to sure choices, error choices and decision times. We produced two novel predictions that can be tested in future experiments in order to falsify the model. We also explored the input parameter of the model and its relation to the attractor landscape. This exploration showed that, in the uncertain option task, accuracy must be traded for flexibility, in order to benefit from the “sure” option.

### Multiple choice decision or metacognition?

A fundamental consequence of our model is the interpretation of the uncertain option task and related neural activity as stimulus response association instead of metacognitive processing. The ability of the uncertain option task to selectively reflect metacognition has been already questioned in the past [14, 15, 38, 39]. These criticisms are based on simple models that interpret the uncertain option task as a multiple choice decision-making. These models are able to reproduce the behavioral results but they use an abstract description level and for this reason they are unable to account for neurophysiological results. We presented a biologically detailed model that is able to reproduce both the behavioral and the neurophysiological data. Our model, as previously proposed ones, also embeds the hypothesis of a multiple choice decision process. Therefore we can confirm that a multiple choice decision is sufficient to solve an uncertain option task showing behavior comparable to that of monkeys performing the task and neural activity comparable to that experimentally observed [3]. Metacognition is not a precisely defined term, even though multiple definitions have been proposed (see e.g. [14, 39, 40]). Without entering into the debate about metacognition in non-human animals, we note that the current model only implements a three choice decision process that can in principle be implemented by animals with very simple nervous systems.

The X-pattern is a characteristic modulation that is usually associated with decision confidence. This association was first noted by Vickers [28] who reports it as a prediction of his accumulators model, in agreement with the experimental observations of lower confidence reports in error compared to correct responses [30]. More recently, behavioral and neurophysiological results in the rat have been presented related to this X-pattern [1]. They found that the probability of restarting a trial (that is believed to be a proxy of low confidence) increased as a function of difficulty in correct responses and decreased in error responses. They also found the same modulation in the firing rate of orbitofrontal cortex neurons. In another recent study [2] it was found that the time the subject is willing to wait for reward (another supposed proxy of confidence) presents an X-pattern. In the model presented here the X pattern arises as a consequence of the probabilistic choice dynamics. We note that, while confidence is usually considered as an instance of metacognition, the model presented here implements a simple multiple choice decision mechanism. Finally, we want to remark that the X pattern is not a necessary condition for confidence since it has been shown that confidence reports in humans can follow different patterns [31–33].

### Other models

Since many models have been proposed for confidence processing and even specifically for the uncertain option task, we want to briefly discuss and compare them here.

The first and most deserved comparison is with the continuous attractor model [9]. The behavioral and neural data recorded experimentally [3] have been already explained by this model. The model is a biologically detailed neural network implementing a ring attractor mechanism. The connections of excitatory neurons are tuned so that each cell fires preferentially to a given direction of motion. Excitatory neurons are placed on a ring according to their direction selectivity. This model was first proposed [41] to account for data of a multiple choice decision-making experiment [21]. However Wei and Wang [9] don’t conclude that experimental data are explained by a decision-making process but that the decision-making process was able to assess the confidence in the decision. The ring model is similar to the one proposed here in the underlying ideas (multiple choice decision model) and modelling framework (attractor network) but they differ in the specific mechanism (continuous *versus* discrete) and predictions. The discrete mechanism that we propose induces an attractor landscape, hence a dynamical system, different from that proposed in [9]. In addition we studied the modification of the attractor landscape produced by the input parameter *λ* and were able to link the uncertain option task with the multistable regime. Importantly, Wei and Wang [9] propose that confidence (defined as 1 − P(*S*)) can be encoded by a sigmoid function of the absolute value of difference between the activity of neural pools corresponding to the two choices (i.e. *σ*(|*R*_*L*_ − *R*_*R*_|), where *σ*(*a*) is the sigmoid function). Conversely, in our model the probability of choosing the sure target does not only depend on the distance between L and R pools but on the maximum of the two (see Fig 4B, 4C and 4D for a cartoon illustration of the difference between the two models). This difference may indicate a difference in the way the two models process the stimulus information to produce choice behavior. Interestingly both models proved to be able to account also for multiple choice decisions in primates see [16, 41], showing many points in common and some differences. How these two models are related to each other is yet unclear and may be investigated in a dedicated study. In particular, it would be very valuable to test the predictions of the two models in order to falsify one of the two or both.

Another model that have been used to account for this task is the one proposed in [13]. This model is based on Signal Detection Theory. The evidence for the decision is represented by a stochastic variable and on each trial the decision is taken comparing the sample of evidence with two thresholds: if the sample is smaller than both thresholds, choice “left” is taken; if the sample falls between the two thresholds, the sure target is chosen; if the sample is larger than both thresholds choice “right” is taken. This model similarly to those proposed by [14, 15] is also embedding the hypothesis of a multiple choice decision. Indeed the stimulus is categorized in three classes depending on the strength of evidence. This model is able to reproduce the behavior of subjects in the uncertain option task, in particular it reproduces the increased performance in free choice compared to forced choice trials. However the model is not dynamic and has no neural interpretation, as a consequence it’s unable to reproduce the neural recordings.

## Supporting Information

S1 Fig Attractors and their basins represented in the *ν*_*L*_, *ν*_*R*_ in the delay condition, i.e. *λ* = 0. Attractors are represented by dots and their basins by dashed lines.
(EPS)

S2 Fig Distributions of firing rates in the phase plane for *λ* = 15 Hz. Each panels shows the distribution of firing rates at *T*_*sure*_ onset for a given combinations of stimulus evidence (Δ*λ*) and stimulus duration as indicated in the figure. It can be observed that, at Δ*λ* = 0, while the system state is quite spread at short durations, it rapidly concentrates around the attractors for longer durations. This in turn produces a large variation in the probability of choosing the sure target. In addition the distributions for different values of Δ*λ* are quite similar and indeed the corresponding probabilities of choosing the sure target are similar. Both this features are not consistent with experimental results of [3].
(EPS)

S3 Fig Conditional probability of choosing the “sure” option given the state of decision neurons, P(*S*|*ν*_*L*_, *ν*_*R*_), for different values of *λ*. The general form of P(*S*|*ν*_*L*_, *ν*_*R*_) is not affected by this parameter. Color convention is the same of Fig 4.
(EPS)

S4 Fig A: 1 − P(*S*), that [9] defines as “confidence”, as a function of |*ν*_*L*_ − *ν*_*R*_| shows a sigmoid pattern. B: 1 − P(*S*) as a function of *ν*_*L*_ + *ν*_*R*_ also shows a sigmoid pattern meaning that in our model the activity of both *ν*_*L*_ and *ν*_*R*_ is important to determine P(*S*).
(EPS)

S5 Fig Scatter plot of model against experimental data. A: Scatter plot showing the probability of correct responses in free choice trials, i.e. with sure target. Each color and symbol represent one value of *λ* as indicated in legend. The dashed lines are regression lines for each subset of points according to color. Shaded areas around regression lines show the interval of confidence of regression lines. Pearson correlation coefficients between model and data are indicated using the same color code. B: Scatter plot as in A representing the probability of a correct response in forced choice trials, i.e. without sure target. C: Scatter plot as in A representing the probability of a sure target response.
(EPS)

S6 Fig Decision times distributions for a parameter set that produces a bistable regime (*λ* = 15Hz). Two different stimulus durations are shown in forced choice trials (upper row) and free choice trials (bottom row), as in Fig 8; Δ*λ* = 7 Hz for all panels. The thresold for the decision is 24 Hz. The color of the histogram represents the relative contribution of correct (green) and error (red) trials to the global distribution. The bimodality induced by the multistable regime (Fig 8) disappears here (bistable regime).
(EPS)

S7 Fig Decision times distributions as in Fig 8 varying decision threshold *ϑ* for free choice trials, sure target presented at 1000 ms. The value of *ϑ* is indicated above each panel, *λ* = 55Hz, Δ*λ* = 7 and stimulus duration is 500 ms for all panels.
(EPS)

S8 Fig Decision times distributions as in Fig 8 varying decision threshold *ϑ* for forced choice trials, without sure target. The value of *ϑ* is indicated above each panel, *λ* = 55Hz, Δ*λ* = 7 and stimulus duration is 500 ms for all panels.
(EPS)

S9 Fig Distribution of bootstrap samples (10000 samples) of the number of error trials in fast and slow trials. For each value of Δ*λ* and stimulus duration the histogram of the number of error trials is shown in blue for fast trials and in magent for slow trials. The vertical line represent the number of error trials in network simulations using the same color scheme. The mean of the distribution for slow and fast trials are different for all value of Δ*λ* and stimulus duration (t-test, all p-values smaller than numeric resolution).
(EPS)
